# Circ_0028826 Promotes Growth and Metastasis of NSCLC via Acting as a Sponge of miR‐758‐3p to Derepress IDH2 Expression

**DOI:** 10.1111/crj.13802

**Published:** 2024-08-07

**Authors:** Lihong Guo, Xueqin Liu, Jie Zhang, Zhuixing Liu, Bohao Zhang, Yang Sun, Dandan Cui, Jinpeng Liu

**Affiliations:** ^1^ Department of Oncology Xi'an International Medical Center Hospital Xi'an Shaanxi China

**Keywords:** circ_0028826, circRNA, IDH2, miR‐758‐3p, NSCLC

## Abstract

**Background:**

Non–small cell lung cancer (NSCLC) is one of the cancers with the highest mortality and morbidity in the world. Circular RNAs (circRNAs) are newly identified players in carcinogenesis and development of various cancers. This study is aimed at exploring the functional effects and mechanism of circ_0028826 in the development of NSCLC.

**Methods:**

Real‐time quantitative PCR (RT‐qPCR) was used to detect the expression levels of circ_0028826, IDH2 mRNA, and miR‐758‐3p. IDH2, Bcl2, Bax, and E‐cadherin protein levels were detected using a western blot. Cell Counting Kit‐8 (CCK‐8), 5‐ethynyl‐2′‐deoxyuridine (EdU), flow cytometry, wound healing, and transwell assays were used to assess the capacities of proliferation, apoptosis, migration, and invasion. Interaction between miR‐758‐3p and circ_0028826 or IDH2 was validated using a dual‐luciferase reporter assay. The role of circ_0028826 in vivo was checked based on a xenograft tumor model.

**Results:**

Circ_0028826 was elevated in NSCLC, and its absence inhibited NSCLC cell proliferation, migration, invasion, and induced apoptosis. In terms of mechanism, circ_0028826 increased IDH2 expression by targeting miR‐758‐3p. In addition, circ_0028826 knockdown also regulated IDH2 by targeting miR‐758‐3p to inhibit tumor growth in vivo.

**Conclusion:**

Circ_0028826 promoted the development of NSCLC via regulation of the miR‐758‐3p/IDH2 axis, providing a new strategy for NSCLC treatment.

## Graphical abstract

Circ_0028826 expression was upregulated in NSCLC patients and cell lines, and its knockdown might inhibit proliferation, migration, and invasion and induce apoptosis in NSCLC cells via targeting the miR‐758‐3p/IDH2 axis.

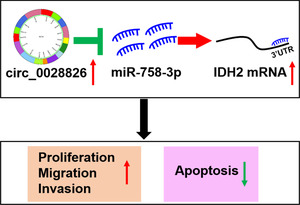

## 1 Introduction

Lung cancer mortality is the highest in the world, and research has determined that non–small cell lung cancer (NSCLC) represents about 80% of all cases [1, 2]. NSCLC has a high potential for invasion and metastasis, and about 75% of patients are already in an advanced stage when they are discovered. Therefore, NSCLC patients have a poor prognosis [3]. Understanding the molecular and cellular mechanisms of some promising biomarkers may provide additional treatment options for NSCLC.

Increasing evidence has suggested that circular RNA (circRNA) is a newly identified player in carcinogenesis and malignant development of various cancers. Different from other noncoding RNAs, circRNA possesses covalently closed‐loop RNA structures formed by connecting 5′ and 3′ terminals [4, 5]. In the past few decades, substantial literature has confirmed that many circRNAs are differently regulated with tissue‐specific expression in cancer tissues and normal tissues [6, 7]. Further studies have indicated that circRNAs are implicated in diverse tumor cell events, such as proliferation, migration, differentiation, and metabolism [8, 9]. As a matter of fact, many circRNAs have been increasingly recognized as a promising biomarker for guiding cancer diagnosis and treatment [10]. However, the role and mechanism of certain novel circRNAs in NSCLC are still unclear. Herein, previous studies in our laboratory have found that circ_0028826, a newly discovered circRNA, is highly expressed in NSCLC, but its function and mechanism of action are still unknown.

There is a growing body of research suggesting circRNAs with miRNA response elements might derepress target mRNA expression by competitively binding to miRNAs at the post‐transcriptional level [11, 12]. It has been reported that multiple combinations of circRNAs and miRNAs present a higher value in diagnosing NSCLC [13]. Of note, isocitrate dehydrogenase 2 (IDH2) is a catalytic enzyme that oxidatively decarboxylates isocitrate to α‐ketoglutarate (α‐KG) [14]. Recently, several cancers have been discovered to be associated with gain‐of‐function mutations of isocitrate dehydrogenase 1/2 (IDH1/2) enzyme [15]. The gain‐of‐function mutation of IDH1/2 has been confirmed to modulate the expression levels of multiple genes and activation of oncogenes [16]. IDH1 and IDH2 catalyzed the oxidative decarboxylation of isocitrate to α‐KG. As the most frequently mutated metabolic gene in human cancers, IDH1/2 interferes with cell metabolism and epigenetic regulation, thereby promoting tumorigenesis [17]. According to reports, wild‐type IDH1/2 participates in the reductive carboxylation process of glutamine, thereby supporting redox homeostasis during anchorage‐dependent tumor formation [18]. For instance, IDH2 converts carboxylate α‐KG from glutamine to citric acid during hypoxia to promote the growth of glioblastoma cells and improve their viability [19]. A recent study has indicated that the upregulation of wild‐type IDH2 boosts NSCLC cell proliferation [20], implying the vital role of IDH2 in NSCLC. Yet, whether IDH2 participates in the circ_0028826 regulatory network is unknown.

In the current research, we first analyzed and characterized a novel circRNA, circ_0028826, and explored whether the circ_0028826/miR‐758‐3p/IDH2 axis was capable of regulating NSCLC development.

## 2 Materials and Methods

### 2.1 Clinical Tissues

NSCLC tumors and matched paracancer samples were respectively isolated from 56 NSCLC patients at Xi'an International Medical Center Hospital and stored under −80°C. Meanwhile, these patients had not received any systemic treatment, including chemoradiotherapy therapy, before sampling. The project was authorized by the ethics committees of this hospital. Before data collection, all subjects provided informed consents.

### 2.2 Cell Culture

The human NSCLC cell lines (NCI‐H1299, A549, SK‐MES‐1, and Calu‐3) and the noncancerous cell line HBE from Ek‐Bioscience (Shanghai, China) were cultured at 37°C with 5% CO_2_ in RPMI 1640 medium (PAN Biotech, Aidenbach, Germany) with 10% FBS (PAN Biotech).

### 2.3 RT‐qPCR

Based on the TRIzol reagent (Sigma‐Aldrich, St. Louis, MO, USA), extracted total RNAs were prepared, and then template DNA was synthesized using the PrimeScript II 1st Strand cDNA Synthesis Kit (TaKaRa, Dalian, China). After undergoing an amplification reaction using a SYBR Green Mix (TaKaRa), samples from different groups were subjected to U6 or β‐actin normalization and 2^–ΔΔCt^ method analysis. Sequences are presented in Table 1.

**TABLE 1:** Primer sequences.

Name		5′‐3′
Circ_0028826	Forward	TCAAGGGTGATGTCACCGAC
	Reverse	AGAAGGTTCTTAGCAGTGGCT
miR‐338‐3p	Forward	GTATGAGTCCAGCATCAGTGA
	Reverse	CTCAACTGGTGTCGTGGAG
miR‐370‐3p	Forward	GTATGAGGCCTGCTGGGGTGG
	Reverse	CTCAACTGGTGTCGTGGAG
miR‐543	Forward	GTATGAGAAACATTCGCGGTG
	Reverse	CTCAACTGGTGTCGTGGAG
miR‐580‐3p	Forward	GCCGAGTTGAGAATGATGAA
	Reverse	CTCAACTGGTGTCGTGGAG
miR‐665	Forward	GTATGAGACCAGGAGGCTGAGGC
	Reverse	CTCAACTGGTGTCGTGGAGTCG
miR‐758‐3p	Forward	GTATGAGTTTGTGACCTGGTC
	Reverse	CTCAACTGGTGTCGTGGAG
miR‐1278	Forward	GCCGAGTAGTACTGTGCATA
	Reverse	CTCAACTGGTGTCGTGGAG
IDH2	Forward	GCCGGCACTTTCAAAATGGT
	Reverse	GATGGACTCGTCGGTGTTGT
β‐Actin	Forward	TGGATCAGCAAGCAGGAGTA
	Reverse	TCGGCCACATTGTGAACTTT
U6	Forward	CTCGCTTCGGCAGCACA
	Reverse	AACGCTTCACGAATTTGCGT

### 2.4 Cell Transfection

Lentivirus expressing circ_0028826 shRNA (sh‐circ_0028826) or sh‐NC (Geneseed, Guangzhou, China) was used to infect NCI‐H1299 and A549 cells. After that, stable cells infected with these lentiviruses were screened according to 1 μg/mL puromycin over 72 h. Oligonucleotides of miR‐758‐3p/in‐miR‐758‐3p and controls from Geneseed and plasmids (Sangong, Shanghai, China), pcDNA, and pcDNA‐IDH2 were transfected into NSCLC cells at 70%–80% confluency, based on Lipofectamine 3000 (Invitrogen, Paisley, Scotland, UK), for 48 h.

### 2.5 CircRNA Subcellular Localization

In this experiment, RNAs from nuclear components and cytoplasmic fractions were distinguished, referring to the Nuclear Extraction Kit (Millipore, Darmstadt, HE, Germany). Then, an RT‐qPCR assay was performed to examine the circ_0028826, β‐actin (cytoplasm control), and U6 (nucleus control) expression in the nucleus and cytoplasm, respectively.

### 2.6 Western Blot

Using a Protein Extraction Kit (Phygene, Fuzhou, China), total proteins were acquired. After quantification based on the Lowry method, samples were subjected to 10%–12% SDS‐PAGE and electrotransferred onto PVDF membranes. Subsequently, immunoblot analyses were implemented via probing with primary antibodies (Abcam, Cambridge, MA, USA) Bcl2 (1:2000, ab182858), Bax (1:1000, ab32503), E‐cadherin (1:10000, ab40772), IDH2 (1:1000, ab131263), and β‐actin (1:50000, ab8227) at 4°C overnight. After being incubated with a secondary antibody for a further 2 h at 37°C, the ECL Reagent Kit (Share, Shanghai, China) was applied to assess the signal of the membranes.

### 2.7 RIP

The relationship between circ_0028826 and miR‐758‐3p was identified in this experiment using a RIP assay kit (MBL, Woburn, MA, USA). In short, NCI‐H1299 and A549 cells were lysed in a complete RIP lysis buffer. Then, cell extract was mixed with Protein A/G Agarose beads (YEASEN) coated with Ago2 or IgG (RNO03M, MBL). After being isolated, coprecipitated RNAs were subjected to RT‐qPCR analysis of circ_0028826 and miR‐758‐3p.

### 2.8 Dual‐Luciferase Reporter Assay

Binding sites between miR‐758‐3p and circ_0028826 or IDH2 3′UTR were predicted based on CircInteractome, starBase, and circBank, followed by being constructed into pmirGLO (Promega, Madison, WI, USA). These constructs were termed circ_0028826‐WT and IDH2 3′UTR‐WT. Meanwhile, site‐directed mutant constructs in the seed sequence (circ_0028826‐Mut and IDH2 3′UTR‐Mut) were provided by the QuikChange Multi Site‐Directed Mutagenesis Kit (Stratagene, La Jolla, CA, USA). When tumor cell confluence reached 70%, these constructs (0.1 μg) and miR‐758‐3p mimic or mimic NC (40 nM) were transfected into NCI‐H1299 and A549 cells using Lipofectamine 3000 (Invitrogen) for 48 h. Finally, the luciferase activities in cell lysates were analyzed using a dual‐luciferase reporter assay system (Promega).

### 2.9 Cell Counting Kit‐8 (CCK‐8) Assay

CCK‐8 (Vazyme, Nanjing, China) was used to detect cell viability. In brief, 1 × 10^4^ A549 or NCI‐H1299 cells were cultured at 37°C for 24, 48, and 72 h, followed by the addition of 10 μL CCK‐8. Four hours later, a microplate reader was applied for measuring the absorbance at 450 nm.

### 2.10 EdU

After being mixed with EdU Solution (RiboBio, Guangzhou, China) for 2 h at 37°C, treated cells were fixed with a 4% formaldehyde solution for 30 min and permeabilized with 0.5% Triton X‐100 for 10 min. After PBS washing, the Apollo reaction cocktail was added to each well, which was then stained with DAPI (identifying the nuclei) for 30 min. At last, the results were analyzed under a fluorescence microscope.

### 2.11 Flow Cytometry Analysis of Cell Apoptosis

After being harvested, treated tumor cells were resuspended in a binding buffer. Following 5 μL Annexin VFITC and 10 μL PI (BD, San Diego, CA, USA) double staining at 4°C in the dark, cell samples were loaded on a flow cytometer and analyzed using FACSDiva software.

### 2.12 Wound Healing Assay

A wound healing assay was utilized for determining the migration ability of A549 and NCl‐H1299 cells. In brief, we seeded A549 and NCl‐H1299 cells into a 12‐well plate. When the density reached 80% following transfection, an Eppendorf tube tip (200 L) was applied to scrape the small linear wound. Then, cells were cultured in serum‐free medium for 24 h. The edges of the scratch were photographed at 0 and 24 h using a microscope (×40, Lcica).

### 2.13 Transwell Assays

In short, 4 × 10^5^ transfected A549 and NCl‐H1299 cells resuspended in RPMI 1640 medium without serum were planted into upper chambers with Matrigel (BD Biosciences, Heidelberg, Germany). Meanwhile, the lower counterpart possessed the medium with 10% FBS for 48 h. After being stained with crystal violet, the outer cells in 10 random fields were imaged under a microscope (Leica).

### 2.14 IHC

After being fixed in 10% formaldehyde and embedded, tumor samples were cut into 4‐μm‐thick sections, followed by incubation with the primary antibody (Ki67 or IDH2) at 4°C for 2 h and the secondary antibody for 1 h. Finally, the slides were counterstained with hematoxylin (Beyotime) and dehydrated, followed by imaging using microscopy.

### 2.15 Xenograft Models

This experiment was permitted under the Animal Welfare and Research Ethics Committee of Xi'an International Medical Center Hospital. 1 × 10^6^ A549 cells with sh‐circ_0028826 or sh‐NC were subcutaneously injected into male BALB/c nude mice (6–8 weeks old, Charles River, Beijing, China, *n* = 5 per group) under specific pathogen‐free conditions. The width (W) and length (L) of tumors were measured weekly, and the tumor volume (V) was calculated according to the following formula: *V* (mm^3^) = *L* × *W* ^2^ × 0.5. Five weeks postinjection, tumor weights were determined after being euthanized using 2% methoxyflurane and cervical dislocation.

### 2.16 Statistical Analysis

Data were shown as means ± standard deviations (SDs) and analyzed using GraphPad Prism. The *p* value in the Spearman correlation analysis was calculated by Spearman's correlation test. *p* < 0.05 was deemed statistical significance. The statistical significance of differences was assessed based on Student's *t*‐tests or one‐way ANOVA.

## 3 Results

### 3.1 Circ_0028826 Was Upregulated in NSCLC

**FIGURE 1:**
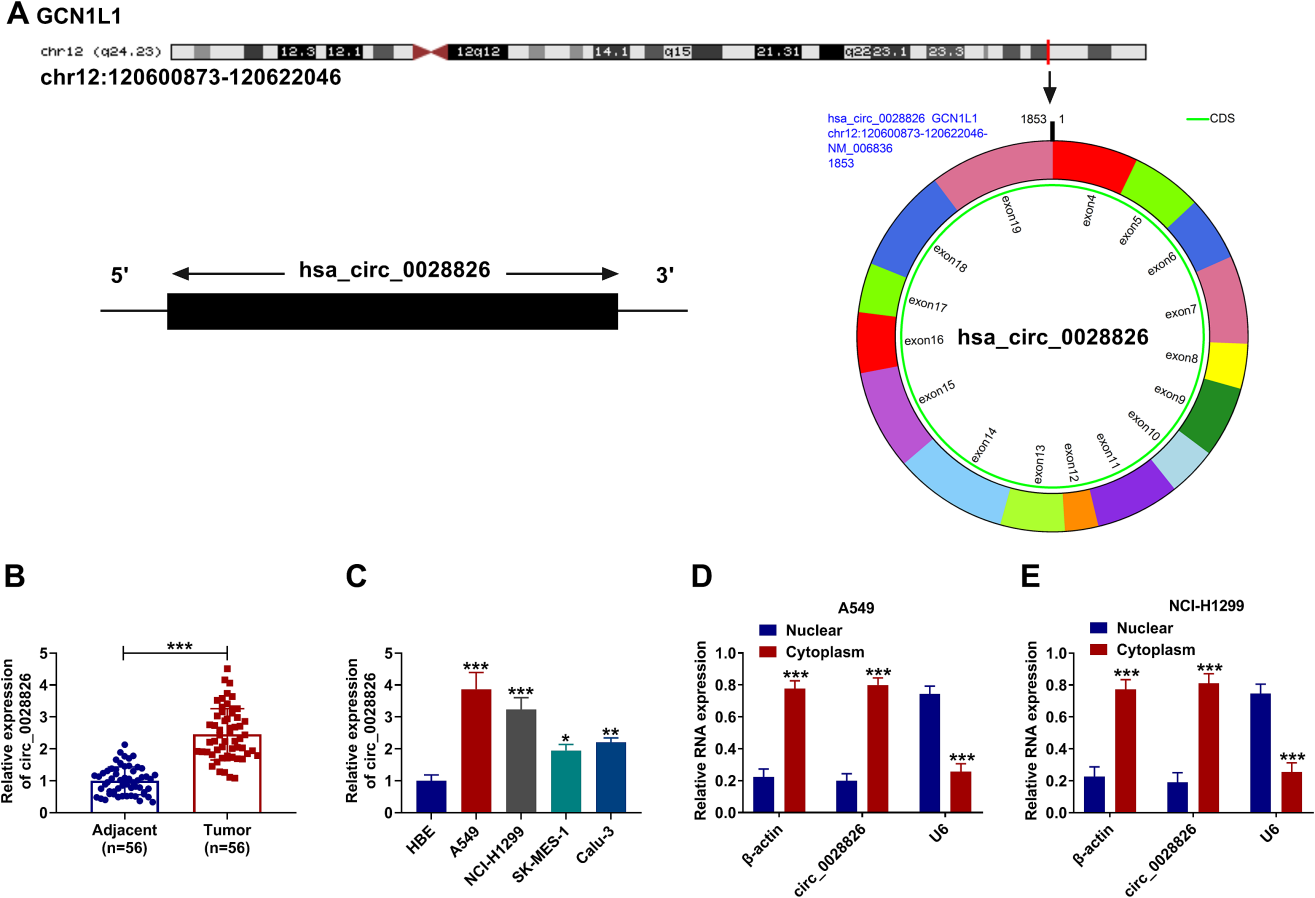
The expression level of circ_0028826 in NSCLC tissues and cells. (A) Circ_0028826 structure diagram. (B, C) The relative expression level of circ_0028826 was analyzed by RT‐qPCR assay in NSCLC tissues and cells, along with in control groups. (D, E) Circ_0028826 was mainly located in the cytoplasm. *p < 0.05, **p < 0.01, ***p < 0.001.

Circ_0028826 arises from the GCN1L1 gene, which is located at chromosome 12 and consists of the back‐splicing of exons 4–19 (Figure 1A). Circ_0028826 expression was higher in human NSCLC tissues compared to corresponding adjacent normal tissues (Figure 1B). We also found that NSCLC cell lines displayed a higher level of circ_0028826 than control HEB cells (Figure 1C). What is more, the circ_0028826 content in A549 and NCl‐H1299 cells was higher than in SK‐MES‐1 and Calu‐3 cells. For this reason, A549 and NCL‐H1299 cells were used for further experimental research. In addition, circ_0028826 was mostly scattered in the cytoplasm of NSCLC cells (Figure 1D,E). In a word, the abnormal expression of circ_0028826 might be associated with the NSCLC process.

### 3.2 Knockdown of Circ_0028826 Might Repress NSCLC Cell Growth and Metastasis

**FIGURE 2:**
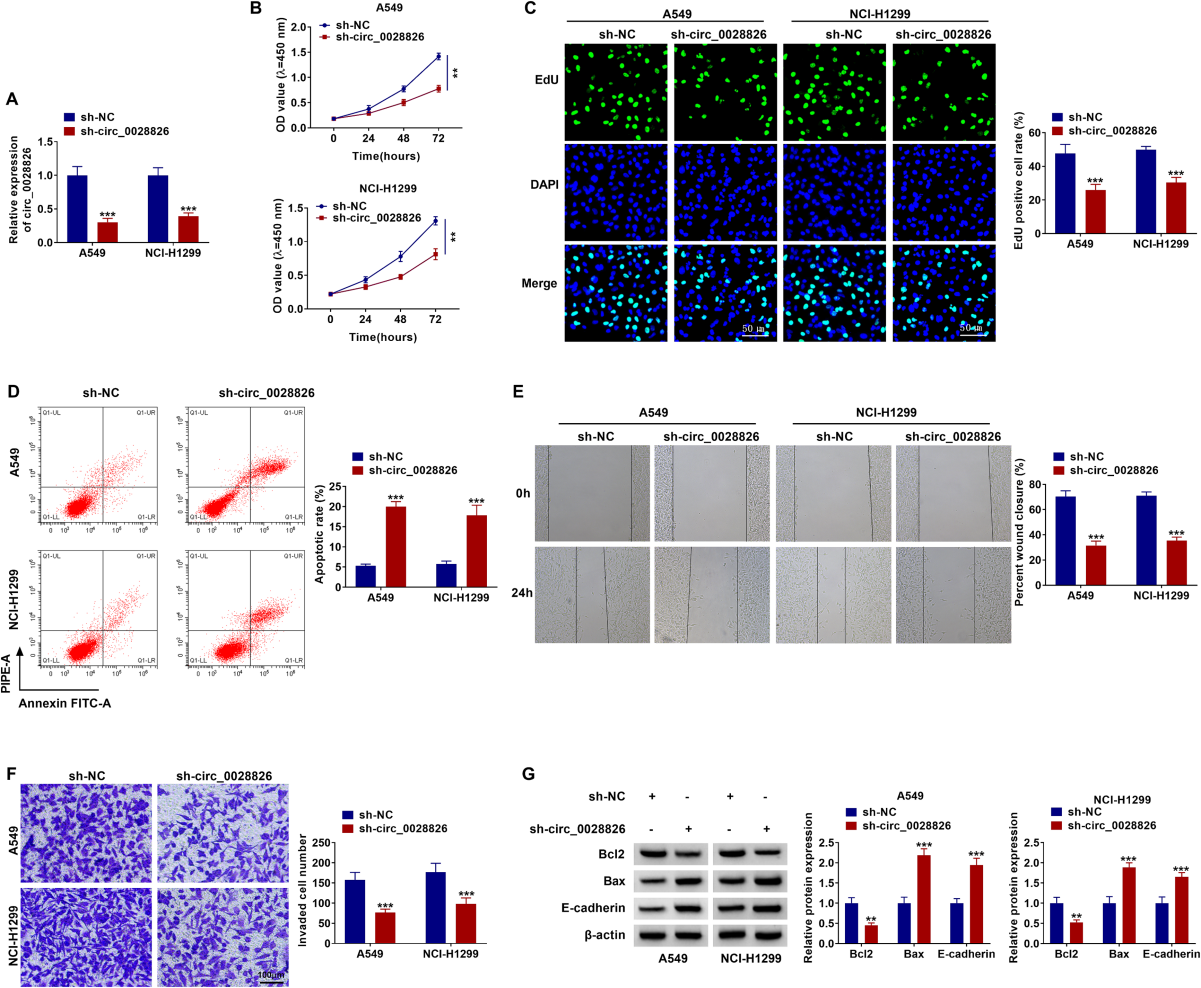
The functional roles of circ_0028826 on cell proliferation, apoptosis, migration, and invasion in NSCLC. (A) The expression level of circ_0028826 in A549 and NCl‐H1299 cells transfected with sh‐circ_0028826 and sh‐NC. (B, C) The cell proliferation was measured by CCK‐8 and EdU assay. (D) Flow cytometry analysis was used to assess apoptosis of transfected A549 and NCl‐H1299 cells. (E, F) The cell migration and invasion were measured by wound healing and transwell assays. (G) Western blot analysis was used to examine the expression of the apoptosis markers (Bax and Bcl2) and the epithelial marker E‐cadherin. **p < 0.01, ***p < 0.001.

As shown in Figure 2A, circ_0028826 expression was significantly diminished in sh‐circ_0028826–transfected NSCLC cell lines (Figure 2A), implying the knockdown is successful. Next, circ_0028826 silencing might hinder the proliferation of both A549 and NCl‐H1299 cells (Figure 2B,C). Furthermore, the downregulation of circ_0028826 might induce the increase of A549 and NCl‐H1299 cell apoptosis rates (Figure 2D). Besides, wound healing and transwell assays showed that the deficiency of circ_0028826 might observably diminish A549 and NCl‐H1299 cell migration (Figure 2E) and invasion abilities (Figure 2F, scale bar, 100 μm). In addition, deficiency of circ_0028826 might decrease Bcl2 protein level (an antiapoptosis marker) and increase Bax expression (a proapoptosis marker) and E‐cadherin expression (a specific marker for erythroid differentiation; increased E‐cadherin might repress cell invasion and migration) in NSCLC cells (Figure 2G). Overall, these data highlighted that circ_0028826 absence might hinder the malignant progression of NSCLC in vitro.

### 3.3 Circ_0028826 Might Directly Bind to miR‐758‐3p in NSCLC Cells

**FIGURE 3:**
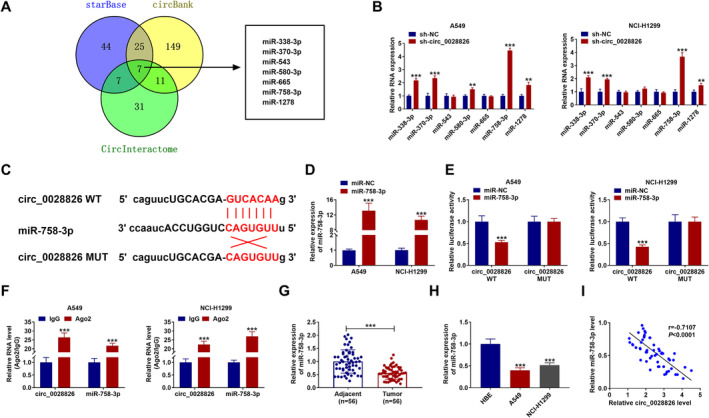
miR‐758‐3p was a direct target of circ_0028826. (A) CircInteractome, starBase, and circBank analyses were carried out to predict the miRNAs that could bind to circ_0028826. (B) Detecting the expression of the seven predicted miRNA in A549 and NCl‐H1299 cells with circ_0028826 knockdown. (C) The complementary sequences between miR‐758‐3p and circ_0028826 were shown. (D) The expression level of miR‐758‐3p was detected by RT‐qPCR after A549 and NCl‐H1299 cells were transfected with miR‐758‐3p or miR‐NC. (E, F) Dual‐luciferase reporter and RIP assays were performed to confirm the association between miR‐758‐3p and circ_0028826. (G, H) The relative expression level of miR‐758‐3p was measured by RT‐qPCR assay in NSCLC tissues and cells. (I) The correlation relationship between miR‐758‐3p and circ_0028826 was analyzed by Pearson's correlation analysis. ***p < 0.001.

To determine the miRNA associated with circ_0028826 in NSCLC, CircInteractome (https://circinteractome.nia.nih.gov), starBase (https://starbase.sysu.edu.cn/), and circBank (https://www.circbank.cn/) were used to predict the miRNAs bound to circ_0028826. Seven miRNAs were eventually predicted by employing Venny 2.1.0 analysis (Figure 3A), which were subjected to RT‐qPCR analysis responding to circ_0028826 knockdown. As a result, the highest level of miR‐758‐3p was observed in tumor cells versus other miRNAs (Figure 3B). Hence, miR‐758‐3p was selected for further research. The binding sites of circ_0028826 and miR‐758‐3p are shown in Figure 3C. Subsequently, miR‐758‐3p content was significantly increased in miR‐758‐3p mimic‐transfected A549 and NCl‐H1299 cells (Figure 3D), implying that overexpression efficiency is available. Then, the relationship between circ_0028826 and miR‐758‐3p was identified using luciferase reporter and RIP assays. As presented in Figure 3E, miR‐758‐3p addition evidently decreased the luciferase activity of circ_0028826‐WT rather than the mutant group (Figure 3E). Moreover, the RIP experiment displayed the significant enrichment of circ_0028826 and miR‐758‐3p in the anti‐Ago2 group (Figure 3F). Besides, our data exhibited that miR‐758‐3p content in NSCLC tumor tissues and cells was reduced (Figure 3G,H). Furthermore, miR‐758‐3p was negatively correlated with circ_0028826 in NSCLC tissues according to Pearson's correlation analysis (Figure 3I). Overall, circ_0028826 served as a sponge for miR‐758‐3p.

### 3.4 miR‐758‐3p Downregulation Partly Recovered Circ_0028826 Knockdown–Mediated NSCLC Cell Malignant Behavior Repression

**FIGURE 4:**
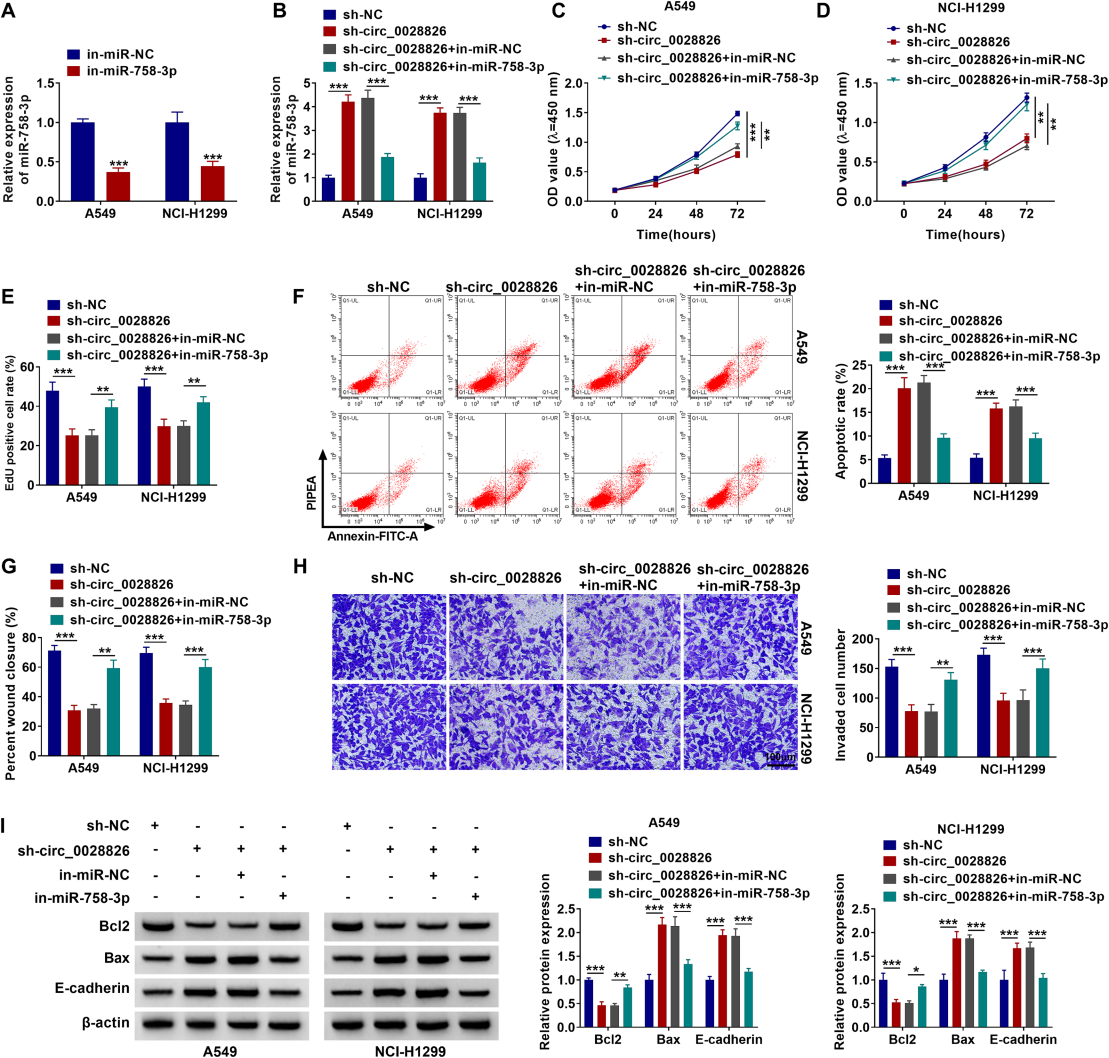
Circ_0028826/miR‐758‐3p axis regulated proliferation, apoptosis, migration, and invasion of NSCLC cells. (A) The expression level of miR‐758‐3p was detected by RT‐qPCR after A549 and NCl‐H1299 cells were transfected with in‐miR‐758‐3p or in‐miR‐NC. (B–I) A549 and NCl‐H1299 cells were transfected with sh‐NC, sh‐circ_0028826, sh‐circ_0028826+in‐miR‐NC, or sh‐circ_0028826+in‐miR‐758‐3p. (B) RT‐qPCR was used to examine miR‐758‐3p level in transfected NSCLC cells. (C–E) CCK‐8 and EdU were conducted to assess cell proliferation in transfected A549 and NCl‐H1299 cells. (F) The cell apoptosis was quantified by flow cytometry assay. (G, H) The cell migration and invasion were measured by wound healing and transwell assays. (I) Western blot analysis was performed to estimate Bcl2, Bax, and E‐cadherin levels. *p < 0.05, **p < 0.01, ***p < 0.001.

The effects of circ_0028826/miR‐758‐3p on NSCLC cell development were further explored. The results showed that the expression of miR‐758‐3p was significantly inhibited in A549 and NCl‐H1299 cells transfected with miR‐758‐3p inhibitors (Figure 4A). Moreover, in‐miR‐758‐3p cotransfection partly counteracted circ_0028826 knockdown–mediated miR‐758‐3p expression promotion (Figure 4B). Functional analysis indicated that deficiency of circ_0028826 significantly suppressed proliferation (Figure 4C–E) and promoted apoptosis (Figure 4F), whereas miR‐758‐3p inhibition abolished these effects in A549 and NCl‐H1299 cells. Lack of miR‐758‐3p reversed circ_0028826 interference‐mediated migration (Figure 4G and Figure S1A,B) and invasion (Figure 4H; scale bar, 100 μm) suppression. Additionally, miR‐758‐3p knockdown could abolish circ_0028826 silencing–triggered Bcl2 protein level reduction and Bax and E‐cadherin protein level enhancement (Figure 4I). In short, these results suggested that in‐miR‐758‐3p recovered the repression of circ_0028826 knockdown on tumor cell growth and metastasis.

### 3.5 IDH2 Was a Direct Target Gene of miR‐758‐3p

**FIGURE 5:**
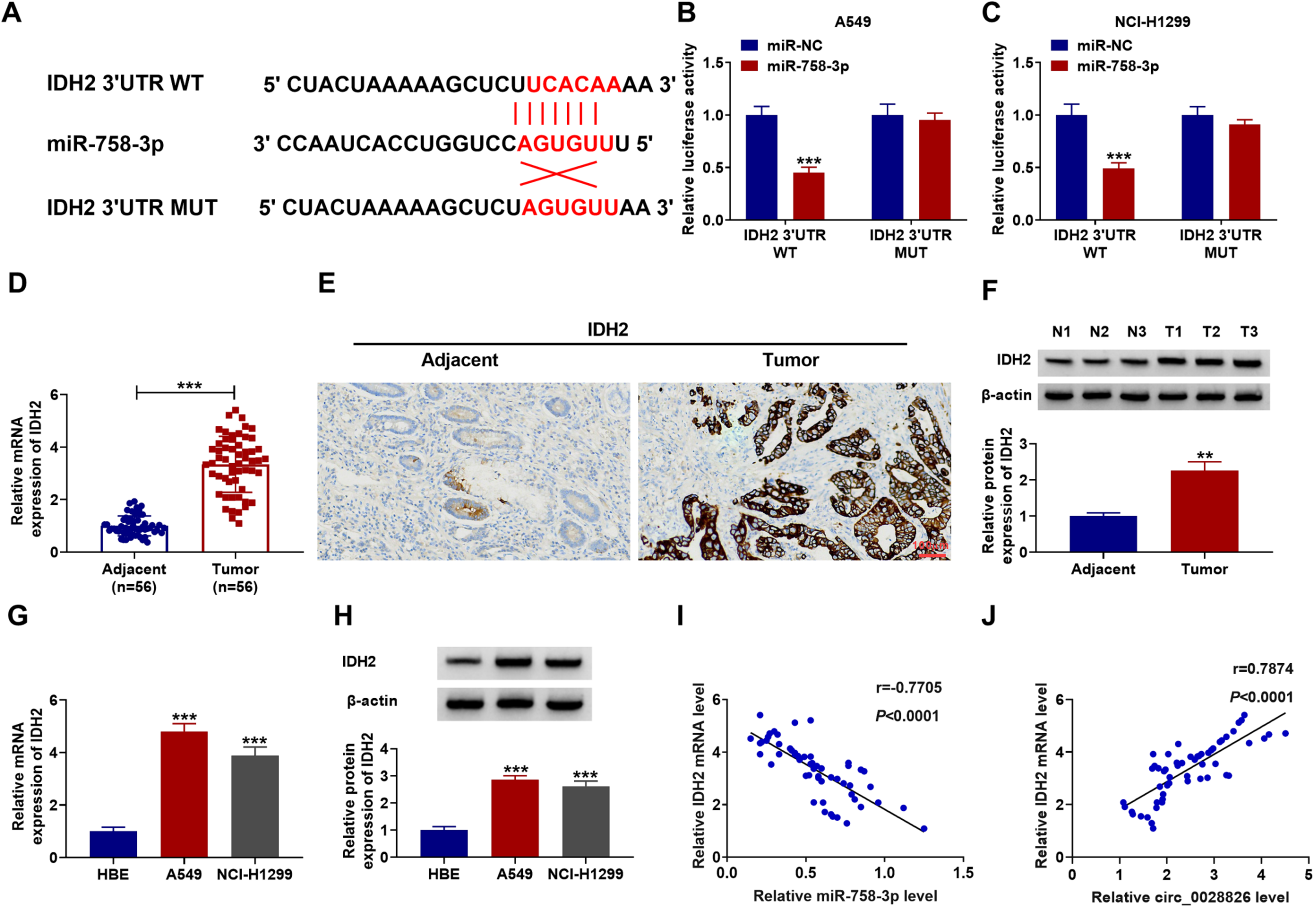
miR‐758‐3p targeted IDH2 in NSCLC. (A) miR‐758‐3p had the binding regions in 3′UTR of IDH2 mRNA. (B, C) Dual‐luciferase reporter was performed in A549 and NCl‐H1299 cells. (D–H) The expression levels of IDH2 were determined by RT‐qPCR, IHC, and western blot analysis in NSCLC tissues and cells. (I, J) The correlation relationships between IDH2 and miR‐758‐3p or circ_0028826 were analyzed by Pearson's correlation analysis. **p < 0.01, ***p < 0.001.

Subsequently, starBase was used to predict the targets of miR‐758‐3p. Potential miR‐758‐3p binding sites were found in IDH2 3′UTR (Figure 5A). Then, a dual luciferase assay showed that miR‐758‐3p overexpression might clearly diminish the luciferase activity of A549 and NCl‐H1299 cells rather than the mutant group (Figure 5B,C). Through RT‐qPCR, IHC (scale bar, 100 μm), and western blot analysis, we observed that IDH2 expression was clearly elevated in NSCLC tumor tissues (Figure 5D–F). Besides, the expression of IDH2 in NSCLC cells was also enhanced according to the data from RT‐qPCR and western blot assays (Figure 5G,H). IDH2 had a negative correlation with miR‐758‐3p and a positive correlation with circ_0028826 in NSCLC tumor tissues by Pearson's correlation analysis. Overall, circ_0028826 targeted miR‐758‐3p to positively regulate IDH2 expression.

### 3.6 IDH2 Overexpression Recovered the Repression of miR‐758‐3p on NSCLC Cell Malignant Behaviors

**FIGURE 6:**
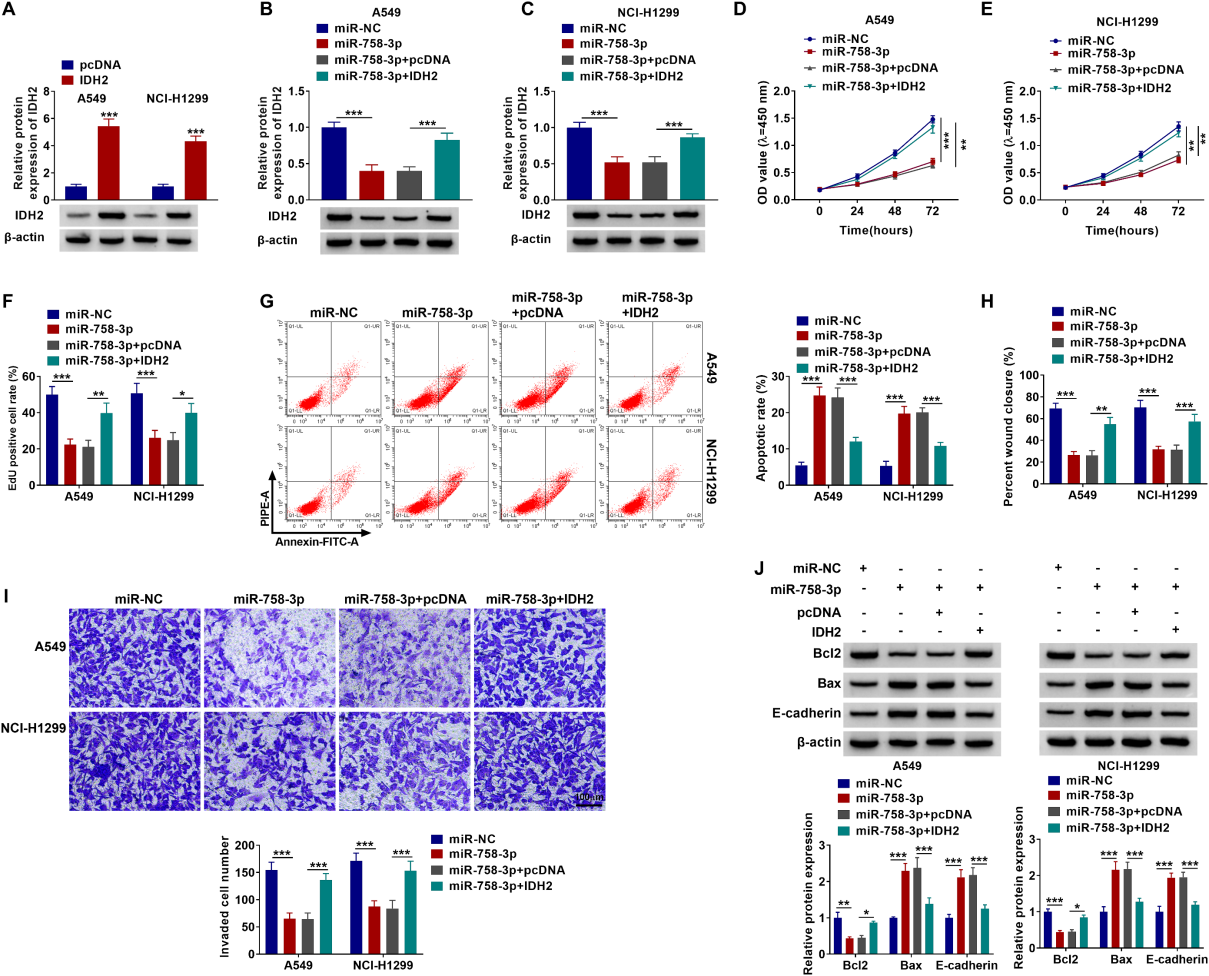
miR‐758‐3p/IDH2 axis regulated proliferation, apoptosis, migration, and invasion of NSCLC cells. (A) The expression level of miR‐758‐3p was detected by RT‐qPCR in A549 and NCl‐H1299 cells transfected with pcDNA or IDH2. (B–J) A549 and NCl‐H1299 cells were transfected with miR‐NC, miR‐758‐3p, miR‐758‐3p+pcDNA, or miR‐758‐3p+IDH2. (B, C) RT‐qPCR and western blot were used to examine IDH2 protein level in transfected A549 and NCl‐H1299 cells. (D–F) CCK‐8 and EdU were conducted to assess cell proliferation in transfected A549 and NCl‐H1299. (G) The cell apoptosis was quantified by flow cytometry assay. (H, I) The cell migration and invasion were measured by wound healing and transwell assays. (J) Western blot analysis was performed to estimate Bcl2, Bax, and E‐cadherin protein levels. *p < 0.05, **p < 0.01, ***p < 0.001.

Rescue experiments were performed to monitor the effects of miR‐758‐3p and IDH2 on tumor progression. First of all, the IDH2 protein level was largely increased in pcDNA‐IDH2‐transfected A549 and NCl‐H1299 cells (Figure 6A), indicating the overexpression is successful. The overexpression of IDH2 can recover the suppressive role of miR‐758‐3p on IDH2 protein levels in A549 and NCl‐H1299 cells (Figure 6B,C). Reduced cell proliferation caused by miR‐758‐3p mimic was partly overturned by the enforced expression of IDH2 (Figure 6D–F). miR‐758‐3p addition obviously increased apoptosis rates in both A549 and NCl‐H1299, whereas IDH2 overexpression abolished these effects (Figure 6G). Cell migration (Figure 6H and Figure S1C,D) and cell invasion (Figure 6I; scale bar, 100 μm) were blocked by miR‐758‐3p, which were largely ameliorated through oe‐IDH2 cotransfection. Furthermore, Bcl2 protein level was lessened in miR‐758‐3p‐transfected cells, and Bax and E‐cadherin expressions were increased in miR‐758‐3p‐transfected cells, but all of them were largely recovered in miR‐758‐3p+IDH2 cotransfected cells (Figure 6J). Overall, miR‐758‐3p upregulation blocked NSCLC development by suppressing IDH2.

### 3.7 Silencing Circ_0028826 Inhibited NSCLC Tumor Growth In Vivo

**FIGURE 7:**
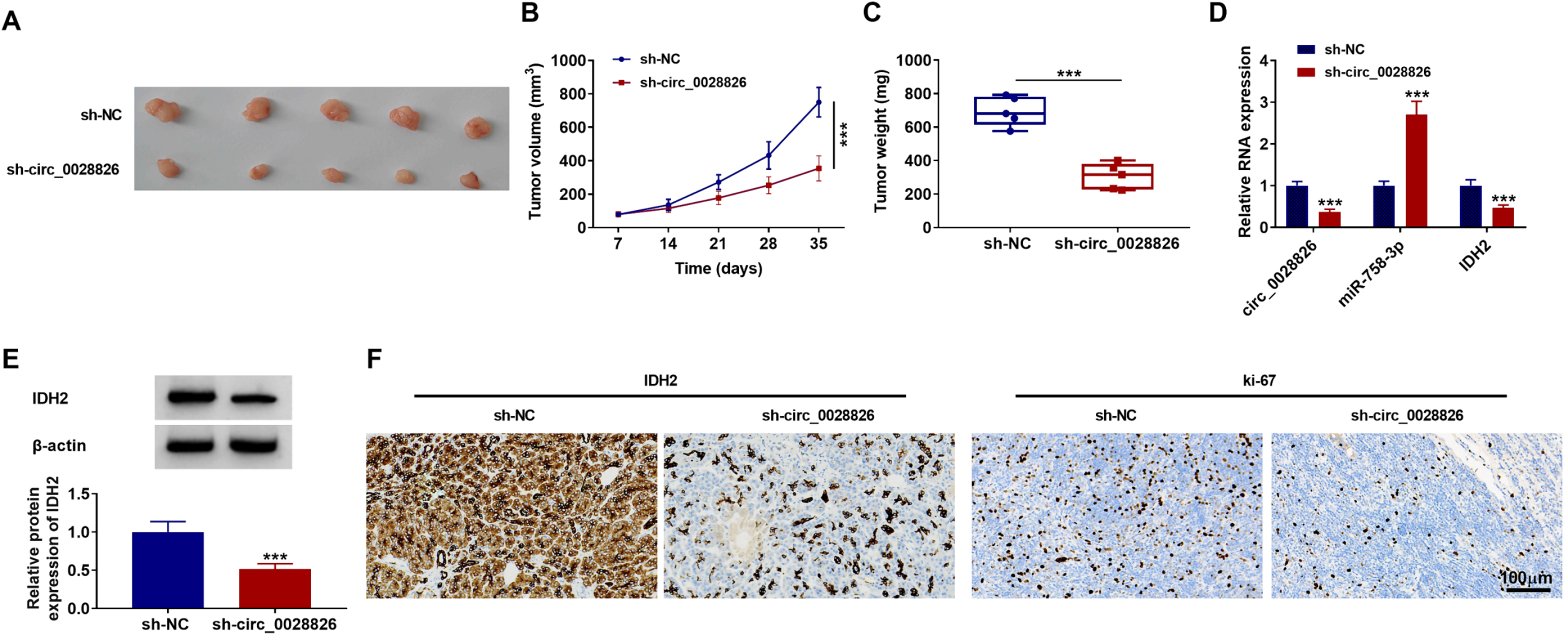
Suppression of circ_0028826 repressed tumor growth in vivo. (A) Tumors were collected and photographed when the mice were killed. (B) After inoculation, tumor volume was recorded once a week. (C) Tumor weight was measured when the mice were killed. (D) The expression of circ_0028826, miR‐758‐3p, and IDH2 mRNA in these excised tumor tissues was measured by RT‐qPCR. (E) The expression of IDH2 at the protein level in these tissues was detected by western blot. (F) IHC analysis was performed to measure the protein expression of IDH2 and Ki67 in tumors from different groups. ***p < 0.001.

NSCLC xenograft models were assayed in vivo to further investigate the roles of circ_0028826. A549 cells with sh‐circ_0028826 were injected subcutaneously into nude mice and allowed to grow for 5 weeks. Data exhibited that circ_0028826 knockdown could markedly decrease tumor volume and weight compared with the negative controls (Figure 7A–C), leading to a smaller tumor size. Furthermore, circ_0028826 and IDH2 levels were decreased, and the miR‐758‐3p level was elevated in tumor tissues from the sh‐circ_0028826 group (Figure 7D,E). Additionally, IHC staining found that the positive expression rates of IDH2 and Ki67 were apparently reduced in tumor tissues derived from sh‐circ_0028826–transfected A549 cells compared with the sh‐NC groups (Figure 7F; scale bar, 100 μm). Together, the circ_0028826 knockdown might block tumor growth in vivo.

## 4 Discussion

Lung cancer is an aggressive cancer, and most of which belongs to the NSCLC [21]. Despite the use of a range of advanced techniques in clinical treatment, the prognosis of NSCLC is still poor due to the atypical early symptoms and the metastasis and recurrence in the majority of patients. Given their critical roles in cancer development, circRNAs are becoming a hot subject in most cancer research [22]. Many circRNAs are recognized as oncogenic drivers in NSCLC. Silencing circ_0067934 inhibits the development of NSCLC [23]. Circ_0016760 upregulation might boost NSCLC cell malignant phenotypic behaviors via the miR‐1287/GAGE1 axis [24]. Herein, a novel identified circRNA, circ_0028826, was validated to be upregulated in NSCLC tissues and cell lines. Functionally, the absence of circ_0028826 significantly blocked NSCLC cell growth and metastasis in vitro. Consistently, circ_0028826 absence–mediated tumor growth repression was proved using in vivo studies. On the whole, these results provided the first‐hand evidence for the oncogene role of circ_0028826 in NSCLC.

Previous reports have indicated that circRNAs might derepress mRNA content through competitive binding to miRNAs. For example, Xiao et al. confirmed that miR‐758‐3p downregulation alleviated circ_0002483 interference‐caused suppression of acute myeloid leukemia [25]. Here, we first identified that circ_0028826 directly targeted miR‐758‐3p. Functional experiments discovered that circ_0028826 absence–mediated NSCLC tumor cell growth and metastasis were partially abolished through miR‐758‐3p inhibition. Interestingly, miR‐758‐3p was verified as a target of circ_0028826, and miR‐758‐3p also existed with a special binding site on IDH2 3′UTR. IDH1/2 is the most frequently mutated metabolic gene in human cancers and interferes with cell metabolism and epigenetic regulation, thereby promoting tumorigenesis. Previous studies discovered that the IDH2 protein was enhanced in lung cancer cells [26, 27, 28]. In our research, IDH2 content was elevated in NSCLC. Beyond that, miR‐758‐3p inhibition might partially overturn sh‐circ_0028826–mediated IDH2 content reduction in NSCLC cells. Furthermore, rescued experiments validated that IDH2 overexpression reversed NSCLC cell growth and metastasis repression caused by miR‐758‐3p. The current data demonstrated that circ_0028826 affects IDH2 content via sponging miR‐758‐3p, supporting the circ_0028826/miR‐758‐3p/IDH2 regulatory network in NSCLC. Nevertheless, there are still some shortcomings in this research. First, considering the limitations of our sample size, more samples from different regions were needed to confirm our results. Second, it is also possible that circ_0028826 might modulate NSCLC development by other mechanisms, such as protein binding, which requires further investigation.

## 5 Conclusion

Herein, circ_0028826 was downregulated in NSCLC tissues compared with normal tissues. Silencing circ_0028826 inhibited NSCLC cell malignant behaviors partly through the miR‐758‐3p/IDH2 pathway modulator. Our data was the first to exhibit the functional effects of circ_0028826 in NSCLC and defined that targeting circ_0028826 might be a treatment strategy for NSCLC.

## Author Contributions

Lihong Guo, Xueqin Liu, and Jinpeng Liu designed the research. Lihong Guo and Xueqin Liu performed the experiments and wrote the manuscript. Jie Zhang and Zhuixing Liu collected the data and made a statistical analysis. Bohao Zhang and Yang Sun are responsible for the software use and graph plotting. Dandan Cui checked the language and grammar. All authors read and approved the final manuscript.

## Ethics Statement

Written informed consents were obtained from all participants, and this study was permitted by the Ethics Committee of Xi'an International Medical Center Hospital.

## Consent

All authors gave their consent for publication.

## Conflicts of Interest

The authors declare no conflicts of interest.

## Supporting information


**Figure S1** Wound healing representative images showing the effect of circ_0028826, miR‐758‐3p, and IDH2 on NSCLC cell migration.

## Data Availability

The data that support the findings of this study are available from the corresponding author upon reasonable request.
