# Supplementary figures and images for: Circ_0028826 Promotes Growth and Metastasis of NSCLC via Acting as a Sponge of miR‐758‐3p to Derepress IDH2 Expression
Source: Clin Respir J. 2024 Aug 7;18(8):e13802. doi: 10.1111/crj.13802 (PMC11306285; doi:10.1111/crj.13802)

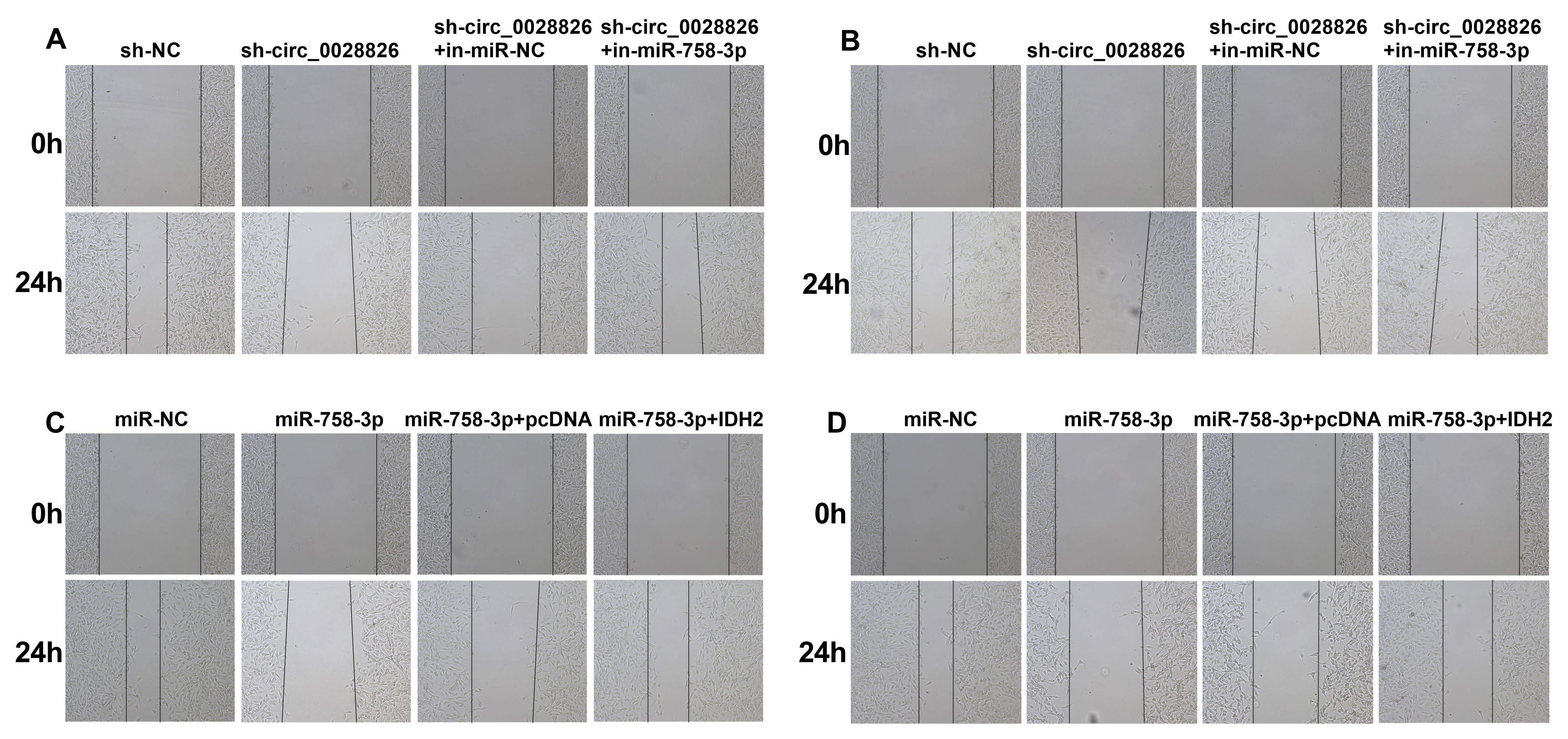

Supplement: Supplementary file 1 — Figure S1 Wound healing representative images showing the effect of circ_0028826, miR‐758‐3p, and IDH2 on NSCLC cell migration. [file CRJ-18-e13802-s001.tif]
